# Supplementary material for: Fast sequence-based microsatellite genotyping development workflow
Source: PeerJ. 2020 May 4;8:e9085. doi: 10.7717/peerj.9085 (PMC7204839; doi:10.7717/peerj.9085)
Supplement: Figure S1 [file peerj-08-9085-s002.docx]

Supporting Figure S1 : Effect of sequence coverage (usuing one, two or three Illumina Miseq nano flowcells) on SSRseq data quality for *Alosa* species.
